# Participant Engagement in and Perspectives on a Web-Based Mindfulness Intervention for 9-1-1 Telecommunicators: Multimethod Study

**DOI:** 10.2196/13449

**Published:** 2019-06-19

**Authors:** Darragh C Kerr, India J Ornelas, Michelle M Lilly, Rebecca Calhoun, Hendrika Meischke

**Affiliations:** 1 Department of Health Services University of Washington Seattle, WA United States; 2 Department of Psychology Northern Illinois University DeKalb, IL United States; 3 Northwest Center for Public Health Practice University of Washington Seattle, WA United States

**Keywords:** occupational stress, occupational health, mental health, mindfulness, telecommunications

## Abstract

**Background:**

Demanding working conditions and secondary exposure to trauma may contribute to a high burden of stress among 9-1-1 telecommunicators, decreasing their ability to work effectively and efficiently. Web-based mindfulness-based interventions (MBIs) can be effective in reducing stress in similar populations. However, low engagement may limit the effectiveness of the intervention.

**Objective:**

The aim of this study was to assess participant engagement in a Web-based MBI designed for 9-1-1 telecommunicators. Specifically, we sought to describe the following: (1) participant characteristics associated with intervention engagement, (2) participant perspectives on engaging with the intervention, and (3) perceived challenges and facilitators to engaging.

**Methods:**

We used qualitative and quantitative data from participant surveys (n=149) that were collected to assess the efficacy of the intervention. We conducted descriptive and bivariate analyses to identify associations between demographic, psychosocial, and workplace characteristics and engagement. We conducted a thematic analysis of qualitative survey responses to describe participant experiences with the MBI.

**Results:**

We found that no individual participant characteristics were associated with the level of engagement (low vs high number of lessons completed). Participant engagement did vary by the call center (*P*<.001). We identified the following overarching qualitative themes: (1) the participants perceived benefits of mindfulness practice, (2) the participants perceived challenges to engage with mindfulness and the intervention, and (3) intervention components that facilitated engagement. The participants expressed positive beliefs in the perceived benefits of practicing mindfulness, including increased self-efficacy in coping with stressors and increased empathy with callers. The most commonly cited barriers were work-related, particularly not having time to participate in the intervention at work. Facilitators included shorter meditation practices and the availability of multiple formats and types of intervention content.

**Conclusions:**

The findings of this study suggest that efforts to improve intervention engagement should focus on organizational-level factors rather than individual participant characteristics. Future research should explore the effect of mindfulness practice on the efficiency and effectiveness of 9-1-1 telecommunicators at work.

**Trial Registration:**

ClinicalTrials.gov NCT02961621; https://clinicaltrials.gov/ct2/show/NCT02961621

## Introduction

### Background

9-1-1 telecommunicators listen to the worst events of our lives, experiencing a slice of a traumatic moment alongside us. Although 9-1-1 telecommunicators are not present at the scene, they may experience secondary trauma. These stressors, coupled with the unpredictability of calls and limited control over the events they are witnessing, may lead to emotional distress [1,2]. Exposure to secondary trauma and work-related stress in 9-1-1 telecommunicators has been associated with posttraumatic stress disorder (PTSD) [3,4], acute stress disorder [5], secondary traumatic stress [6], and occupational burnout [5,6]. The costs of work-related stress extend beyond the individual telecommunicator, including decreased productivity [7], increased absenteeism [7], and increased health care utilization and expenditures [8,9]. With the lives of others depending on the ability of 9-1-1 telecommunicators to work effectively and efficiently, there is a significant need for stress-reduction interventions within this population.

Mindfulness-based interventions (MBIs) have been shown to reduce stress in general populations [10,11]. MBIs encourage participants toward a nonjudgmental acceptance of stressors through the cultivation of present-centered awareness [12-14]. Web-based MBIs, delivered via the internet or a computer portal, have been shown to have comparable effect sizes on stress as in-person formats [15]. Furthermore, Web-based MBIs may be particularly well suited for the 9-1-1 telecommunicator population in which shift work and variable schedules make in-person training difficult. However, low engagement and high dropout rates in Web-based MBIs and other Web-based health promotion interventions can limit intervention effectiveness [16-19]. Several studies and meta-analyses of Web-based interventions have reported a dose-response relationship in which increased engagement is associated with better outcomes [19-21]. An understanding of who engages and how they engage with Web-based MBIs is important to ensure that the interventions are reaching those who may need them most, to maximize engagement and to improve the effectiveness of future interventions. This necessitates a multimethod approach that assesses effective engagement, or “sufficient engagement to achieve the intended outcomes,” rather than quantitative measures of engagement alone [22].

Studies have indicated that a paradoxical relationship may exist, in which the psychological symptoms that MBIs intend to ameliorate may make it more difficult for participants first engaging in mindfulness practice to attain present-centered awareness [23-26]. Thus, MBI participants may become discouraged and disengage from the intervention [26]. Stress [24], low trait mindfulness [25], PTSD [25], and perseverative thinking [23,26] have been proposed as factors that may hinder engagement in mindfulness practice. In addition, overcommitment, a perseverative thinking style marked by “excessive striving and a strong need for approval and esteem at work” may make it more difficult for some participants in workplace MBIs to engage [1,27]. Conversely, a commitment to practice and to incorporate mindfulness into daily life, as well as positive beliefs in the benefits of practicing mindfulness, may encourage engagement [13,28,29]. This study sought to explore how such factors influence engagement in MBIs.

Workplace MBIs may have additional barriers or facilitators to participant engagement. Supportive work environments may offer social support, which is a facilitator of engagement [18,30-32]. Factors outside of the control or measure of the intervention, such as staffing policies or the workplace physical environment, may also influence engagement.

### Objectives

This study is an analysis of several aspects of participant engagement in a Web-based workplace MBI designed to reduce stress and increase mindfulness in 9-1-1 telecommunicators. In addition to behavioral outcomes (the outcome paper is under review), the randomized controlled trial included process measures related to engagement and qualitative survey data collected throughout the intervention. The aims of this study were to use both the process data and qualitative survey data to explore the following: (1) measurable factors associated with the level of engagement (low vs high) in the intervention, (2) participant perspectives on engaging with the MBI, and (3) the perceived challenges and facilitators to engagement in general.

## Methods

### Study Design and Recruitment

This study was an analysis of participant engagement in the treatment arm of a randomized controlled trial (ClinicalTrials.gov NCT02961621), testing the effectiveness of a Web-based MBI to reduce stress among 9-1-1 telecommunicators [33]. The outcomes of the efficacy study are currently under review.

Study recruitment was conducted in 2 phases. First, emergency response call centers in the United States and Canada were recruited using industry publications and email announcements. To be eligible for the study, the call centers had to allow their employees to receive emails and use the internet to access the intervention website. The enrolled call centers (n=31) represented rural, urban, and suburban areas in the United States and Canada and responded to 9-1-1 calls for police, fire, or medical emergencies or a combination of call types. In the second phase of recruitment, individual 9-1-1 telecommunicators were recruited from within the enrolled call centers. Recruitment differed at each call center but relied mainly on staff announcements, recruitment emails and flyers, and word-of-mouth. Individual participants were required to be currently employed as a 9-1-1 telecommunicator (ie, call-receivers, dispatchers, or both). A total of 323 telecommunicators (n=325 were assessed for eligibility; n=2 declined to participate) were enrolled in the study [33].

The study was approved by the Institutional Review Board of the University of Washington. After obtaining electronic informed consents, the participants provided demographic and employment-related (ie, length of employment) information and completed a Web-based baseline survey [33].

### Web-Based Mindfulness Intervention

The Web-based mindfulness intervention was modeled after Mindfulness-Based Stress Reduction, which has been shown to be effective for a variety of physical and mental conditions [10,11,33-35]. Clinicians and investigators trained in mindfulness developed the intervention to meet the specific needs of 9-1-1 telecommunicators. After consultation with stakeholders at the enrolled call centers, intervention developers adapted the intervention content from the traditional in-person format to an abbreviated Web-based format to address logistical concerns.

**Figure 1:**
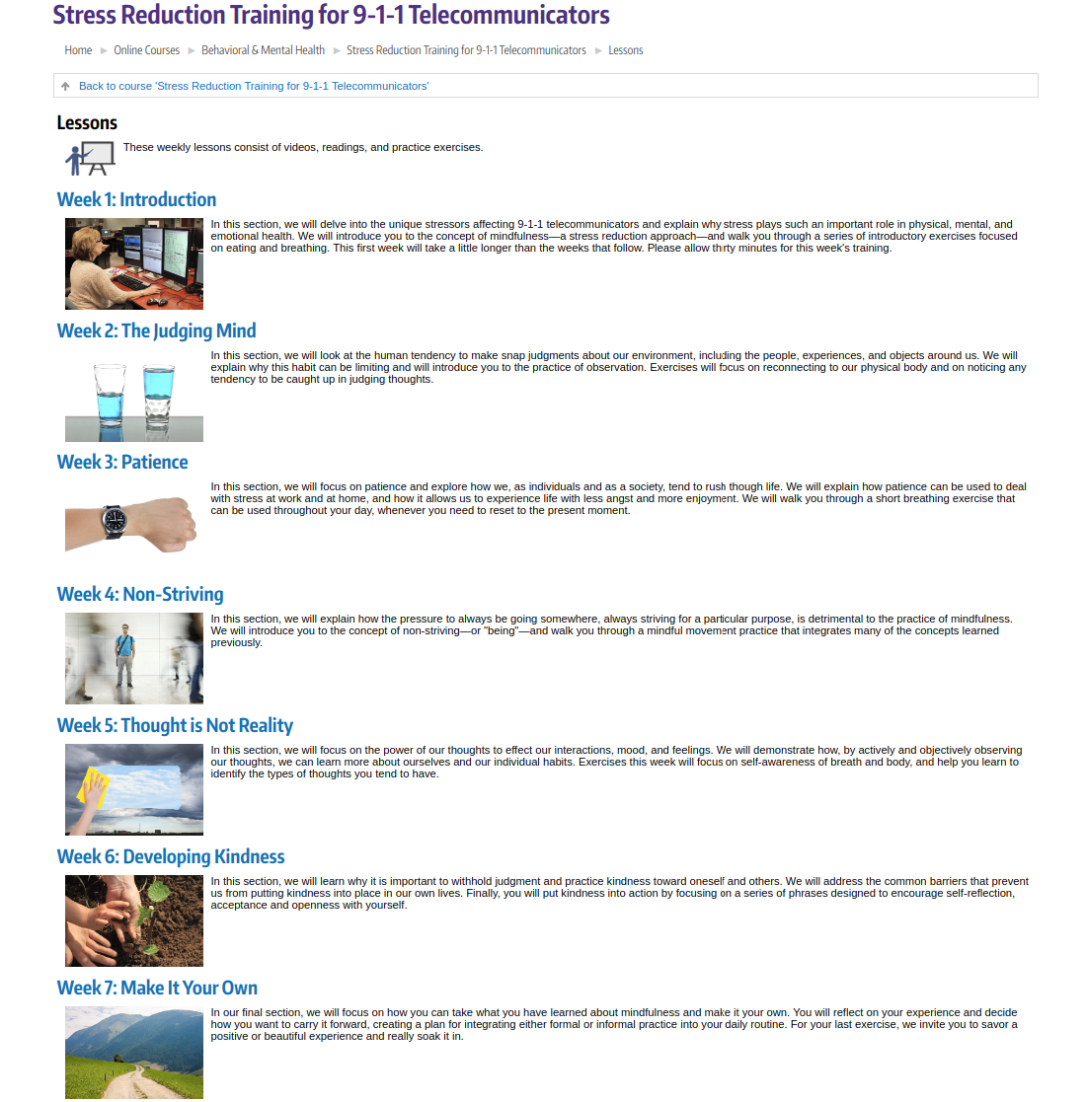
Overview of weekly lessons.

**Figure 2:**
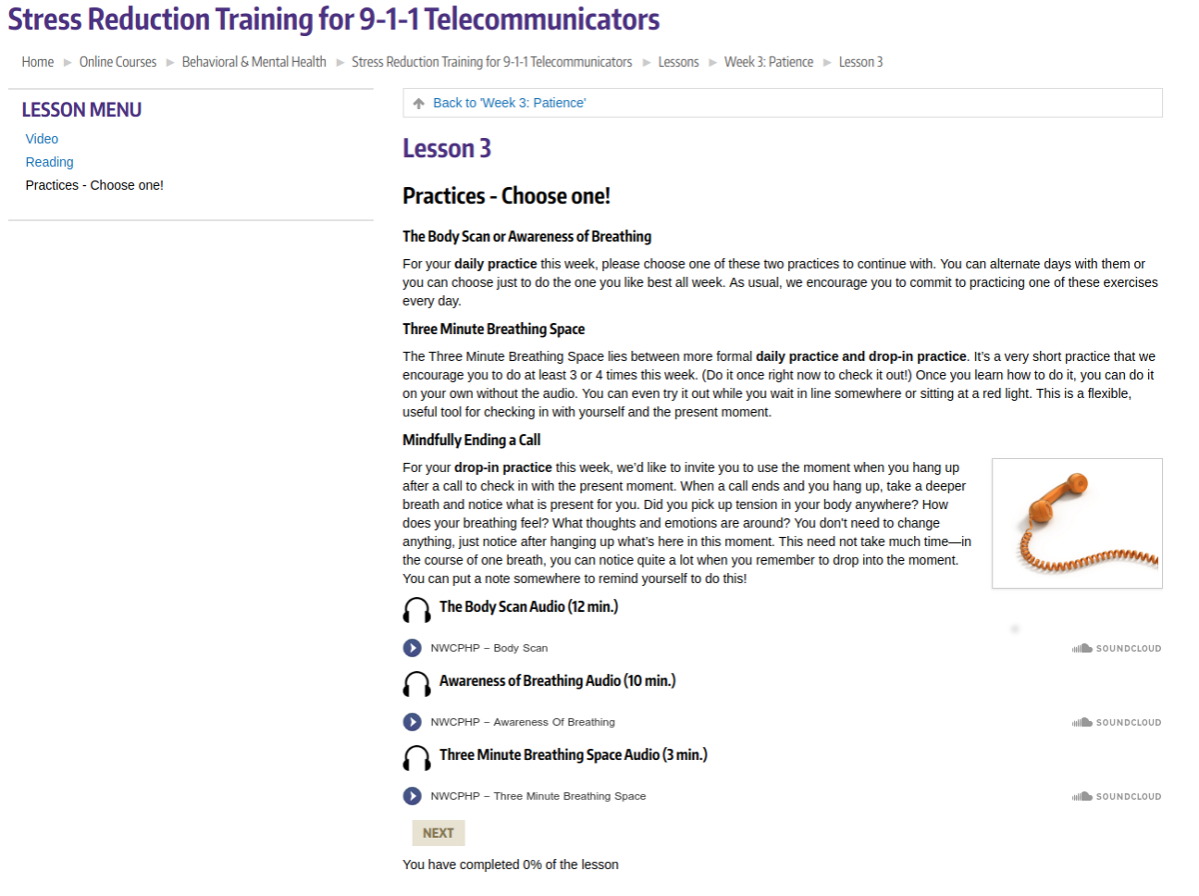
Suggested daily and drop-in practices for 1 lesson.

The intervention’s 7 Web-based lessons were hosted on the learning management system of the Northwest Center for Public Health Practice at the University of Washington (Figures 1 and 2). Each lesson started with a short video that introduced that week’s theme and was followed by a short reading. The next section of the lessons consisted of 1 longer (10 to 14 min) *daily practice* with guided audio that introduced formalized meditation skills, such as body scan and loving-kindness, and 1 to 2 brief *drop-in* mindfulness practices focused on incorporating mindfulness activities into daily life. Some of these practices, such as *body awareness at your desk* and *mindfully ending a call*, were tailored specifically for the emergency response call center environment. Each lesson also included a weekly check-in survey and an optional moderated discussion board. The estimated time to complete each lesson was between 20 and 30 minutes [33].

### Intervention Procedures

After completion of a baseline survey, the participants randomized to the intervention (N=161) were contacted twice weekly throughout the intervention period. One email contained a link to the weekly training lesson, whereas the second email provided suggestions for incorporating mindfulness skills into daily life.

Call center managers were highly encouraged to provide the study participants with a designated time during work to complete the intervention. Participants were asked to complete 1 lesson per week over a 7-week period and were encouraged to complete the lessons on a designated weekday as their work schedules allowed. However, lessons from previous weeks could be accessed throughout the intervention period. Participants were instructed to do the *daily practice* with guided audio for approximately 10 minutes for at least 6 out of 7 days a week and were encouraged to do the *drop-in* practices as often as they were able to.

At the beginning of each weekly lesson, intervention participants were asked to complete Web-based weekly check-in surveys with a mix of close- and open-ended questions. These surveys assessed how often the participants practiced formal mindfulness during the previous week, if and how they incorporated mindfulness into their daily lives, and any perceived effects. There were 6 weekly check-in surveys in total. A final training evaluation after the last weekly lesson (week 7) assessed participant satisfaction, perceived effects, and overall experience. Textbox 1 lists the open-ended questions.

Web-based follow-up surveys identical to the initial baseline survey were completed at 2 weeks and 4 months after the end of the intervention. All the participants received a certificate of completion, and the Washington State Criminal Justice Training Commission: Telecommunicator Program recognized the training as continuing education for renewal of telecommunicator certification [33].

### Measures

#### Main Outcome

##### Engagement

Engagement in the intervention was measured as the number of lessons completed, assessed via access logs for viewing lesson videos. Owing to the small number of participants per lesson completed, we categorized participants into 2 groups: those who completed 0 to 4 lessons (low engagement) and those who completed 5 to 7 lessons (high engagement).

##### Demographic

Participants were asked to self-report their age, gender, race, ethnicity, marital status, highest level of education, years of experience as a 9-1-1 telecommunicator, and whether, currently, they had children under the age of 18 years.

**Open-ended survey questions used in qualitative analysis.**

Weekly check-in survey

1. Can you give 1 or 2 examples of how you incorporated mindfulness into your daily life this week?
2. Please share anything you have noticed this week about the effects of your mindfulness practice.

Final training evaluation

1. What did you like about this training?
2. What effect has this training had on your stress level, if any?
3. What did you dislike or what would you change about the training?
4. Is there anything else you would like to share with us about your experience with this training?

##### Psychosocial

###### The Calgary Symptoms of Stress Inventory

Stress was measured using the 56-item Calgary Symptoms of Stress Inventory, which assesses the frequency of experiencing subjective symptoms of stress with 8 subscales. Participants rate the frequency of experiencing the stress-related symptoms on a 5-point Likert scale from *never* to *frequently* during the past week [36]. Previous research has established the reliability of the instrument in a sample of 9-1-1 telecommunicators, and internal consistency of the scale in the main outcome study was strong (alpha=.95) [1, personal communication by H Meischke, December 5, 2018].

###### Mindful Attention Awareness Scale

Mindfulness was measured using the 15-item Mindful Attention Awareness Scale (MAAS), which assesses mindfulness as a unidimensional construct of attentional awareness in the present moment. Participants are asked how frequently or infrequently they have each stated the experience of mindlessness, conceptualized as the inverse of mindfulness, on a 6-point Likert scale from *almost always* to *almost never* [37]. The MAAS has been used in previous research with 9-1-1 telecommunicators, and the internal consistency of the scale in the main outcome study was strong (alpha=.91) [1, personal communication by H Meischke, December 5, 2018].

##### Work-Related

###### Swedish Demand-Control-Support Questionnaire Social Support Subscale

The 6-item social support subscale of the Demand-Control-Support Questionnaire (DCSQ) was used to assess overall workplace atmosphere and social support from coworkers and supervisors. Participants are asked to report their agreement with statements on a 4-point Likert scale ranging from *strongly disagree* to *strongly agree* [38]. Previous research has established good internal consistency, construct validity, and criterion validity for the English version of the total DCSQ scale (alpha=.83) and good internal consistency for the social support subscale (alpha=.84) [39].

###### Social Support Visual Analog Scale

A visual analog scale (VAS) was also used to measure the overall level of satisfaction with social support in the workplace. The scale ranges from 0 to 100 with 0 representing *completely dissatisfied with social support at work* and 100 representing *completely satisfied with social support at work*. The scale has been employed in previous research in the emergency responder population [40].

###### Network Conflict Visual Analog Scale

A VAS was used to measure the perceived degree of conflict in the individual participant’s workplace social network. The scale ranges from 0 to 100 with 0 representing *little or no conflict with coworkers* and 100 representing *frequent and intense conflict with coworkers*. The scale has been employed in previous research in the emergency responder population [40].

###### Effort-Reward-Imbalance Overcommitment Subscale

The 6-item overcommitment subscale of the effort-reward-imbalance (ERI) is used to measure an individual’s tendency to engage in a coping pattern of excessive commitment and the high need for approval at work [41]. Responses are indicated on a 4-point Likert scale with higher scores indicating greater overcommitment. The subscale has sound psychometric properties, including satisfactory confirmatory factor analysis, internal consistency, and reliability [42]. The ERI has been employed in previous research with 9-1-1 telecommunicators, which found good internal consistency for the overcommitment subscale (alpha=.85) [1].

### Data Analysis

We used a complementary integration of qualitative and quantitative methods to suit each inquiry in this analysis [43]. We employed 2 research paradigms, quantitative positivist and qualitative interpretivist, and have provided a convergent interpretation of the results in the Discussion section [44].

#### Quantitative Data Analysis

Quantitative methods were used to describe demographic, psychosocial, and workplace characteristics associated with the intervention engagement. Statistical analyses were conducted using the R statistical package version 3.4.3 [45]. Bivariate associations between the independent variables (ie, demographic, psychosocial, and workplace characteristics) and participant engagement (ie, low vs high engagement) were tested using chi-square or Fisher exact tests for categorical variables and unpaired *t* tests for continuous variables.

#### Qualitative Data Analysis

Qualitative methods were used to describe the participants’ perspectives of the intervention and to identify perceived barriers and facilitators. A thematic analysis was conducted using Web-based responses to 6 open-ended questions from 6 weekly check-in surveys and a final training evaluation (Textbox 1) [46]. In total, 822 open-ended responses from 109 unique participants at 26 call centers were analyzed. The analysis was conducted without the use of the high-low engagement characterization used in the quantitative analysis.

The first author read the entirety of the data to become familiar with the content and then independently developed an initial coding schema through a hybrid deductive-inductive approach [47]. Deductive coding was used to create an initial codebook that was derived from Banerjee et al’s 5-facet framework for psychological engagement (ie, motivation, intention, commitment, belief, and a therapeutic relationship between the individual, teacher, and group) [23]. On the basis of the content of the survey questions, only 2 out of the 5 facets, commitment and belief, were included in the initial codebook. Meanwhile, inductive coding allowed for the inclusion of themes outside the bounds of the theoretical framework. After creation of the pilot codebook based on the deductive approach, additional broad code categories and subcodes were added based on an initial read-through. A second coder unaffiliated with the study was trained on the coding schema; then the 2 coders independently piloted the schema to establish an initial agreement on how to apply the codes. The 2 team members met iteratively to verbally negotiate any discrepancies during coding and to ensure credibility of the analysis. The first coder identified major themes, and the second coder reviewed the themes to ensure credibility. Sample quotations were identified to illustrate each theme.

## Results

### Participant Characteristics and Engagement

Of the 161 participants enrolled in the intervention’s treatment arm, 12 (7.5%) were excluded from this analysis owing to loss of employment or not completing the baseline survey. Table 1 portrays the sample characteristics of the participants in this analysis (*n*=149). Nearly half of the participants (71/149, 47.7%) completed all 7 intervention lessons whereas 21.5% (32/149) did not complete a single lesson. The mean number of lessons completed was 4.7 (SD 2.8). The mean number of days the participants reported practicing formal mindfulness with guided audio (3 [SD 0.15]) did not differ by engagement group (low vs high) and remained consistent through the intervention.

Of all the participants, 35.6% (53/149) were characterized as low engagement (0 to 4 lessons complete) and 64.4% (96/149) as high engagement (5 to 7 lessons complete). The comparison between low and high engagement showed that demographic, psychosocial, or work-related characteristics were not significantly related to engagement (data not shown). Only the call center of employment was statistically significantly associated with engagement (*P*<.001).

### Participant Perspectives on the Mindfulness-Based Intervention

A total of 3 overarching themes were identified: (1) the participants perceived benefits of mindfulness practice, (2) the participants perceived challenges to engaging with mindfulness and the intervention, and (3) intervention components that facilitated engagement. Textbox 2 provides an outline of the themes and subthemes.

#### The Participants Perceived Benefits of Mindfulness Practice

This theme describes the perceived benefits of engaging in mindfulness practice. Overall, the participants indicated that with continued practice and incorporation of mindfulness into their daily life, practicing mindfulness became easier and the benefits of practice became more perceptible. Consequently, many of the participants indicated that they were more able and willing to continue:

...[mindfulness is] becoming easier to incorporate into my everyday. The more I practice it, the more I want to do it as I am noticing it’s helpful to relax.

##### Mindfulness Helps 9-1-1 Telecommunicators Cope With Stress

The participants frequently reported uncertainty of a direct effect on their stress level; instead, many noted that practicing mindfulness positively changed how they coped with stressors. One participant expressed:

I am not suddenly Zen, but I feel I can identify stress quicker and shake it off easier.

Many of the participants reported that although their stress was still present, they felt more aware, accepting, and in control of how their stress affected their mental and physical well-being:

It's almost like the stress is still hovering there, but I can choose to not think about the stress.

These responses indicate a positive belief in not only the benefits of mindfulness practice but also in one’s ability to apply the concepts of mindfulness to reduce stress.

##### Mindfulness Helps 9-1-1 Telecommunicators Communicate With Callers and Focus at Work

Many of the participants reported that mindfulness enabled them to have more empathy with callers:

I have been trying to change how I think of our callers that frustrate me. Instead of thinking that they are all stupid, I am trying to think they are being silly, or to be more empathetic.

**Table 1:** Characteristics of participants.

Variables			Participants (N=149)^a^
**Demographic characteristics, n (%)**			
	**Age (years)**		
		Below 26	11 (7.4)
		18 to 35	50 (33.6)
		36 to 45	51 (34.2)
		46 to 55	28 (18.8)
		56 to 64	9 (6.0)
	**Gender**		
		Female	126 (84.6)
		Male	23 (15.4)
	**Race**		
		American Indian or Alaska Native	7 (4.3)
		Asian	1 (0.6)
		Black	4 (2.4)
		Multiracial	7 (4.3)
		Native Hawaiian or Pacific Islander	0 (0.0)
		Other	5 (3.0)
		White	141 (86.0)
	**Binary race**		
		Nonwhite	8 (5.4)
		White	141 (94.6)
	**Ethnicity**		
		Hispanic	4 (2.8)
		Non-Hispanic	141 (97.2)
	**Experience as a 9-1-1 telecommunicator (years)**		
		Less than 2	20 (13.5)
		2 to 10	68 (45.9)
		11 to 20	42 (28.4)
		Above 20	18 (12.2)
	**Married or living with a partner**		
		Yes	97 (65.5)
		No	51 (34.5)
	**Children under the age of 18 years**		
		Yes	67 (45.0)
		No	82 (55.0)
	**Highest education**		
		High school or General Education Diploma	12 (8.1)
		Some college	63 (42.3)
		Associates	14 (9.4)
		Bachelors	51 (34.2)
		Postgraduate study or degree	9 (6.0)
**Psychosocial characteristics, mean (SD)**			
	Stress (Calgary Symptoms of Stress Inventory)		56.0 (27.5)
	Mindfulness (Mindful Attention Awareness Scale)		4.1 (0.8)
**Work-related characteristics, mean (SD)**			
	Social support (VAS^b^)		67.8 (24.0)
	Social support (Demand-Control-Support Questionnaire subscale)		17.6 (2.9)
	Network conflict (VAS)		33.3 (25.0)
	Overcommitment (effort-reward-imbalance subscale)		13.4 (3.9)

^a^Some frequencies do not sum up to 149 owing to missing responses.

^b^VAS: visual analog scale.

**Overview of qualitative findings: themes and subthemes.**

Theme 1: The participants perceived benefits of mindfulness practice

- Mindfulness helps 9-1-1 telecommunicators cope with stress
- Mindfulness helps 9-1-1 telecommunicators communicate with callers and focus at work
- The participants perceived broad benefits of mindfulness in their everyday life

Theme 2: The participants perceived challenges in engaging with mindfulness and the intervention

- Stress and mindlessness make it difficult to attain present-centered awareness
- Aspects of the call center environment make it difficult to engage

Theme 3: Intervention components facilitated engagement

- Shorter mindfulness practices were easier to engage with and use at work
- Variety in intervention content allowed participants to choose what worked best for them

Some reported that mindfulness improved their overall communication on the job:

When you approach someone from a place of sincere kindness, it is harder for them to be rude, angry or hostile. I talked to many people on the phones this week very escalated and upset with the police response time, and by using this approach and using my breath to remain aware and present, and non-reactive, I was able to establish better rapport with these callers.

In addition, some of the participants reported that practicing mindfulness improved their focus at work, especially when multitasking. As one participant stated:

In our career we have to multitask all the time...it can be easy to overlook something. I tried to put more of my focus into the audible while still multitasking but noticed I was catching things clearer.

##### The Participants Perceived Broad Benefits of Mindfulness in Their Everyday Life

Participants described how being mindful improved their overall quality of life. Feeling calm was one of the most commonly reported perceived benefits of practicing mindfulness. Many participants attributed this sense of calm to an increased acceptance of external situations. As one participant described it:

I feel calmer. I try not to fret about things that I cannot change.

Many participants also noted that practicing mindfulness made them feel relaxed, energized, and happier. For some of the participants, bringing present-centered awareness into their daily routines made them feel less rushed, thus increasing their appreciation of everyday life. One participant expressed that:

Rather than rushing from task to task I became more present and slowed down, and enjoyed the process of even mundane tasks like folding laundry or putting away groceries.

Some participants noted that practicing mindfulness expanded their self-awareness and increased their sense of control over thoughts:

I think it has made me more aware of how I’m feeling and how my feelings are affecting me both physically and mentally.

I feel more in control of what I’m thinking, acknowledging the randomness of some thoughts.

Participants frequently reported the perceived consequences of practicing mindfulness on their interpersonal relationships as well. Many participants described becoming more patient and less emotionally reactive, for example:

I was able to have a difficult conversation with a family member (parent) much more calmly and less emotionally than in the past. I feel it was different this time because of the mindfulness practices.

I'm finding myself to be way less reactive, I'm more serene and calm, and less sensitive. I feel I'm rolling with the punches and not letting emotional or angry callers get a rise out of me.

In addition, some participants noted that practicing mindfulness improved the quality of their relationships with loved ones:

My kids are excited to tell me about their day because I’m taking more time to focus (that’s hard for me sometimes) and really hear them. They love seeing me excited about “their” day.

Finally, participants frequently reported using mindfulness to cope with physical stressors. Most commonly, participants described practicing mindfulness to fall asleep. Some reported that this helped them not only to fall asleep more quickly but also improved the quality and quantity of sleep.

##### The Participants Perceived Challenges to Engage With Mindfulness and the Intervention

This theme describes the perceived individual, workplace, and intervention-related challenges to engage with mindfulness and the intervention. Although these challenges may not have led the participants to stop using the intervention, they may have made it more difficult for the participants to engage.

###### Stress and Mindlessness Make it Difficult to Attain Present-Centered Awareness

Although many participants described using mindfulness to cope with stressors, some participants described being in a stressful state as a challenge to practicing mindfulness:

[Practicing mindfulness] was difficult this week as my stress and anxiety level was much higher this week...I tried to be mindful as a way of reducing that, but it was difficult.

Being in a stressful state made it more difficult for some participants to attain present-centered awareness. As one participant described it:

I still find during stressful events at work I lose this feeling of awareness and hours can go by where I haven’t focused on my breathing or awareness once.

Some participants described difficulty in disengaging from habitual mindlessness. For example:

I still find that I am going through the motions of my daily activities without thinking about them. Reflection is an afterthought.

However, this may represent a growing awareness of mindfulness and present-centered thinking rather than a challenge to it.

###### Aspects of the Call Center Work Environment Make it Difficult to Engage

Some participants described physical and psychological aspects of the work environment that made it difficult to engage with the intervention, especially with the guided audio content. Frequent interruptions, noise levels, shared workspaces, and busy working conditions were often cited:

I did not like the mindfulness audio. I was not able to use it while at work due to how busy it was, and it was much easier to practice it without the audio on my own time.

Some of the participants posited that aspects of the work environment may have limited the effectiveness of the training:

It's a difficult training to get done in our center due to shared computers and busy shifts. It's not possible to do the segments in our center without interruption and I think this causes it not to be as effective.

Participants from nearly 50% of call centers in the intervention reported not having a designated time-off to complete the intervention despite the intervention protocol. This was a major barrier for completing the intervention content, and some participants believed the intervention increased rather than reduced their stress. For example:

I am finding it is almost causing more stress trying to find the time to get practice in and to do the weekly lessons. We do not have the staffing to permit us time off the floor to complete training, so we must do it while on duty on the floor.

Some participants reported completing the intervention lessons while actively working or while at home. As one participant reported:

It was difficult to listen to the longer listening exercises at our desks while still answering calls and radio traffic. At times, there were too many interruptions that I would get frustrated and just do it at home.

A few participants expressed a belief that mindfulness was incongruous with their work as a 9-1-1 telecommunicator:

When it is busy on the dispatch floor, there really isn’t time to stop and do anything for yourself for the calls that stress you out. Dispatching isn’t that kind of job.

I think that with our job, we do so much multitasking that it becomes hard to focus on just one thing.

#### Intervention Components Facilitated Engagement

This theme describes aspects of the intervention that the participants indicated made it easier for them to engage.

##### Shorter Mindfulness Practices Were Easier to Engage With and Use at Work

Many participants described shorter-length practices as easier to engage with than longer practices. This was often attributed to difficulty in maintaining present-centered awareness during longer practices:

I found it hard to concentrate for the longer exercises, like 12-14 minutes. I know [Name of Instructor] said it was okay to wander, that is part of the process, but I wandered a lot - and kept checking the timer.

Shorter practices were described as “an attainable goal” in comparison with longer practices. Many of the participants reported difficulty in finding the time to do the longer practices at work owing to frequent interruptions. Similarly, some of the participants felt that they would be more able to use shorter practices at work:

We are used to short breaks, always being in a rush... short 5-minute meditations are much more realistic and practical.

##### Variety in Intervention Content Allowed Participants to Choose What Worked Best for Them

Some participants noted that they enjoyed having a variety of content formats (ie, audio, video, and written) and types of practice (eg, loving-kindness and body scan) to try each week. The availability of different content allowed participants to find what worked best for them. As one participant shared:

I liked that there was a variety of practices to try. Different things work for different people and that was taken into account.

## Discussion

### Principal Findings

The goal of this study was to explore the engagement of participants in a Web-based workplace MBI for 9-1-1 telecommunicators with the aims of (1) investigating demographic, psychosocial, and workplace characteristics associated with level of engagement (low vs high) in the intervention, (2) exploring participants’ perspectives on engaging in the intervention, and (3) identifying perceived challenges and facilitators to engaging in the intervention. Results of the quantitative analysis showed that individual-level demographic, psychosocial, or workplace characteristics examined in this study were not associated with engagement. Call center location was the only factor associated with engagement, indicating that workplace-level characteristics rather than individual-level characteristics may be more relevant to intervention engagement. The qualitative results supported this finding; difficulty engaging with the intervention at work was commonly identified as a barrier for participants. In nearly 50% of call centers, at least one participant described not having a designated time to complete the intervention. When developing future MBIs for high-stress work environments, researchers should work closely with the workplace managerial staff to identify potential barriers to employee engagement and develop practical solutions to overcome these barriers. The availability of different formats of intervention materials (eg, written, video, and audio) and shorter practices may be simple mechanisms to facilitate engagement in workplaces that are not able to provide employees time-off to engage with the intervention content.

Although we found no significant association between baseline stress and engagement in the intervention, our qualitative findings suggest that being in a stressful state of mind may make it more difficult to attain present-centered awareness. Moreover, the perception of being time-poor may discourage engagement, especially for workplace interventions that may be perceived as detracting from work time [16]. Although more research is needed on the effectiveness of abbreviated meditation practices, it is nevertheless important to consider that an intervention that is used is more effective than one that is not. Furthermore, more attention should be placed on attaining sufficient engagement to elicit behavior change rather than greater quantities of engagement alone [22]. Brief interventions and short practices may act as stepping-stones to more rigorous practice.

Despite the barriers to engage with the intervention, participants exhibited a high degree of commitment to incorporate mindfulness into their daily lives and expressed positive beliefs in the perceived benefits of practicing mindfulness. Previous findings suggest a positive feedback loop for mindfulness practice in which practice prompts greater perceived benefits, which in turn increases motivation to practice [24]. Although mindfulness is inherently not outcome-focused, future MBIs could highlight the positive benefits of mindfulness practice to encourage engagement.

This intervention’s main outcome study found significant improvements in stress scores postintervention [personal communication by H Meischke, December 5, 2018]. Our qualitative findings help elucidate how participants perceived stress reduction during the intervention. Specifically, participants reported that although their stress was still present, they felt more aware, accepting, and in control of how their stress affected their mental and physical well-being. Mindfulness practice enabled participants to reclassify stress as a changeable rather than a static state of mind while also increasing their efficacy to enact change. These findings suggest that workplace MBIs can provide participants with tools to cope with stressors in a more effective way.

The benefits of mindfulness practice may extend beyond the individual practicing; participants reported that mindfulness increased their focus at work and their empathy with callers thereby improving their overall communications. Future research is needed to determine the effect of mindfulness practice on the efficiency and effectiveness of 9-1-1 telecommunicators. However, this research suggests that mindfulness may be a unique tool for improving communications, emergency or otherwise.

### Strengths and Limitations

A key strength of this study was the use of both qualitative and quantitative methods, which allowed for a richer exploration of intervention engagement than possible with either method alone. Nonetheless, several limitations warrant discussion. Although our study showed a significant difference in participant engagement between call centers and the participants perceived aspects of the workplace as a challenge to engage, we were unable to determine a causal mechanism between organizational-level factors and participant engagement. Additional research using a randomized trial design would inform organizational-level comparisons of participant engagement. Second, we did not have a reliable measure of the intensity of use or attrition. Future studies should include measures that can validly and reliably assess the intensity of use and other process outcomes. Third, the use of Web-based survey data limited the breadth and depth of the thematic analysis. Future qualitative studies using semistructured participant interviews could identify novel themes or add nuance to this study’s findings. Finally, the demographic homogeneity of this study population may limit the external validity of our results.

### Conclusions

This study explored participant engagement in a Web-based workplace MBI among 9-1-1 telecommunicators. Our findings suggest that organizational-level factors may be more pertinent to engagement than individual participant characteristics. Some of the qualitative feedback presented in this study may inform future MBIs in similar high-stress work environments, particularly the need to work closely with managerial staff at intervention sites to mitigate potential barriers to employee engagement. The availability of multiple formats of intervention materials (eg, written, video, and audio) and shorter meditation practices may be simple, cost-effective mechanisms to facilitate engagement. A Web-based workplace MBI can provide emergency responders with tools to cope with stressors, improve general well-being, and build empathy with callers. Future research should explore the effect of mindfulness practice on the efficiency and effectiveness of 9-1-1 telecommunicators at work.

## Abbreviations

DCSQ: Demand-Control-Support Questionnaire
ERI: effort-reward-imbalance
MAAS: Mindful Attention Awareness Scale
MBI: mindfulness-based intervention
PTSD: posttraumatic stress disorder
VAS: visual analog scale
